# Surface Modification of Calcium Silicate via Mussel-Inspired Polydopamine and Effective Adsorption of Extracellular Matrix to Promote Osteogenesis Differentiation for Bone Tissue Engineering

**DOI:** 10.3390/ma11091664

**Published:** 2018-09-09

**Authors:** Chia-Tze Kao, Yen-Jen Chen, Hooi-Yee Ng, Alvin Kai-Xing Lee, Tsui-Hsien Huang, Tz-Feng Lin, Tuan-Ti Hsu

**Affiliations:** 1 School of Dentistry, Chung Shan Medical University, Taichung City 40447, Taiwan; ctk@csmu.edu.tw (C.-T.K.); thh@csmu.edu.tw (T.-H.H.); 2 Department of Stomatology, Chung Shan Medical University Hospital, Taichung City 40447, Taiwan; 3 School of Medicine, China Medical University, Taichung City 40447, Taiwan; yenjenc.tw@yahoo.com.tw (Y.-J.C.); hooiyeen@gmail.com (H.-Y.N.); Leekaixingalvin@gmail.com (A.K.-X.L.); 4 Department of Orthopedics, China Medical University Hospital, Taichung City 40447, Taiwan; 5 3D Printing Medical Research Center, China Medical University Hospital, Taichung City 40447, Taiwan

**Keywords:** calcium silicate, dopamine, bone cement, bio-inspired, tissue engineering, osteogenic

## Abstract

Calcium silicate-based cement has garnered huge interest in recent years, due to its versatility and potential in mass fabrication of a variety of bioceramics. For this study, the main objective was to fabricate functionalized calcium silicate (CS) powder integrated with a simple bio-inspired surface modification using polydopamine (PDA), to regulate cellular behaviors such as cellular adhesion, and subsequently cell differentiation and proliferation. For this study, scanning electron microscopy (SEM) and X-ray photoelectron spectroscopy (XPS) techniques were used to analyze the chemical compositions and observe the surface characteristics of our PDA coated CS cements. Such modifications were found to enhance Wharton Jelly’s mesenchymal stem cells (WJMSC) in various ways. Firstly, PDA-coated CS cements were found to significantly enhance cell adhesion with higher expressions of cell adhesion markers, such as focal adhesion kinase and integrins. This was further supported by morphology analysis of the cells. This enhanced cell adhesion, in turn, led to significantly higher secretion of extracellular matrix (ECM) proteins, such as collagen I and fibronectin, which directly promoted cell attachments and proliferation. In our osteogenesis assays, it was found that secretion and expression of osteogenesis related genes and proteins were significantly higher and were dependent on the PDA content. Therefore, these results demonstrated that such simple bio-inspired modification techniques of synthetic degradable CS cements can be applied as a future modification, to modify and convert inert surfaces of synthetic bone grafts to enhance and modulate the cell behaviors of WJMSCs. This in turn can be used as a potential alternative for further bioengineering research.

## 1. Introduction

Archaeological discoveries suggested that ancient people knew how to harness the bones and teeth of animals or human corpses, to substitute or replace damaged parts of our bones and teeth [1]. Contemporary medical osteotomy, together with the concept of artificial and biocompatible implantations, had since emerged and evolved to become the tissue engineering that we know today. Mineral trioxide aggregates, otherwise known as bone cements, are widely used and applied in bone tissue engineering. Ordinary bone cements have great potential for skeletal tissue reconstruction and production of calcified tissue by stimulating cell proliferation [2]. Amongst the numerous bone cements, the remarkable β-tricalcium phosphate is the most commonly used biomaterial in orthopaedic surgery as a bone graft. This is partly due to the high biocompatibility property that it possesses. However, limitations such as poor biodegradation and osteo-stimulation, have severely limited its complete application in orthopaedic surgery [3]. Thus, scientists intend to explore comprehensive bioactive cements for hard tissue substitution, and one alternative approach is to fabricate composite bone cements. Composites of tricalcium phosphate were formed to compensate for the biodegradability and osteoinductive shortfalls of bone cements.

Therefore, bone tissue engineering is viewed as an alternative and novel strategy to solve issues faced in current bone regeneration research. From the numerous materials available in bone tissue engineering, bone cements are known as a widely used material. Studies have shown that bone cements have excellent bioactivity, which allows a thin apatite layer to be formed rapidly when in contact with simulated body fluid or post-implantation, and evidences have shown that this apatite layer is responsible for the good osteoinduction properties and cell-material adhesion of bone cements [4]. Therefore, bone cements are regarded as one of the most promising candidates in current bone bio-engineering research. Not only do calcium silicate (CS) cements have good bio-activities, but studies have shown that CS cements also have high binding affinities to both live tissues and bones [5]. Much attention has been placed on CS bioactive cements, as compared to calcium phosphate derivatives, due to its versatility. Previous studies have reported that calcium silicate bioactive cements were able to enhance osteogenic and odontogenic differentiation of many different types of stem cells [6,7,8]. There is a requirement for such materials to have a rapid setting time, to prevent inflammation and displacement during the restoration procedure, especially if these materials were to be used and applied in dental surgical procedures [9]. The control of setting time is mainly dependent on the apatite precipitation and mineralization of well-defined biocomposites. Up till now, all attempts to prepare biocomposites with calcium silicate bioactive cements for clinical applications were not very successful, as it is very difficult to achieve sufficient physiological tolerance, biocompatibility, efficient handling properties, and long-term stability, which are of a certain standard for bones and teeth replacement. Such implants were usually unsuccessful in the long run, due to post-surgical infections or toxicity of materials used. However, CS cements are able to release Si ions, which have the ability to influence cellular behaviors of many different cell types [10,11]. These Si ions were found to enhance apatite formation, which greatly enhances the capability of tissue regeneration. A recent study has shown that Si ions inhibited the osteoclastogenic activities of macrophages by directly decreasing the activity of RANKL-enhanced tartrate-resistant acid phosphatase, thereby restricting activation by limiting the NF-κB pathway [12]. Si has also been found to play a part in active calcification of human bone development. In summary, CS cements were found to be able to reduce inflammation, and at the same time enhance osteogenic differentiation of osteoblast and mesenchymal stem cells, thus making CS cements a potential candidate for bone regeneration studies [13,14].

Numerous types of materials including collagen, gelatin, hydroxyapatite, polylactide, and polycaprolactone, were previously used to synthesize composites. A recent novel strategy to enhance the physiological properties of calcium phosphate biocomposites, involves the admixing of biological molecular dopamine (DA) and polydopamine (PDA) into the formulation [15]. This bio-inspired idea is derived from the adhesive strategy of mussels on various hydrophobic or hydrophilic surfaces, which is basically a thin ad-layer film formed by the weak base-triggered oxidation of PDA and polymerization of DA. This novel approach became a new methodology to functionalize a biocompatible substrate via simple chemical processes. It can be used as a simple method to modify characteristics of surfaces, which enhances not only the covalent immobilization of substrates, but it has also been reported to enhance osteogenic differentiation of stem cells [16]. Xu’s study showed that self-assembled biocomposites of Ca-P/PDA nanolayers were able to solve problems of bio-inert scaffolds by improving levels of bioactivity, for application in bone regeneration studies [17]. Furthermore, 3D printed poly(lactic acid) scaffolds were successfully coated with PDA, and were shown to be more functionalized by exhibiting significantly higher cell adhesion and cell proliferation [18].

In our study, we fabricated biocomposites composing of calcium silicate bioactive cements and PDA, to attempt to improve the physiological properties of scaffolds. The bioactivity and biocompatibility of our biocomposites were systematically elucidated by analyzing the adhesion of stem cells, and subsequently its levels of differentiation and growth. Modifying the structural characteristics of biocomposites with a PDA ad-layer would likely be beneficial in improving in vivo cellular bioactivity. In addition, this study aimed to investigate the crystalline structure of biocomposites, based on comparison with the mineral phase of bones and teeth. Various analyses using scanning electron microscopy (SEM), wide angle x-ray diffraction, mechanical testing, and laser scanning confocal microscopy, were applied in this study. As mentioned earlier, such a biocomposites approach may be the way forward for future bone tissue engineering and grafts replacement.

## 2. Materials and Methods

### 2.1. Preparation of PDA-Coated CS

Calcium oxide (CaO), Silicon dioxide (SiO_2_), Aluminum oxide (Al_2_O_3_), and Zinc oxide (ZnO) of reagent grades were purchased from Sigma-Aldrich (St. Louis, MO, USA) and mixed together to a ratio of 65%, 25%, 5%, and 5%, respectively, in terms of weight percentages. This CS with oxide mixture was then heated to a temperature of 1400 °C, and subsequently sintered for 2 h. After which, the end product was ground into a fine CS powder form. Subsequent preparations of PDA-coated CS powder were done using a simple one step immersion technique. For preparation of different concentrations of PDA-coated CS powder, different groups of powder were immersed at 1, 2, and 4 mg/mL in a 10 mM Tris of dopamine (Sigma-Aldrich, St. Louis, MO, USA) calibrated at pH 8.5, at room temperature overnight. The different group codes used in this study, were namely the “DA0”, “DA1”, “DA2”, and “DA4” groups, represented the different concentrations of PDA coating, ranging from 0 to 4 mg/mL. Subsequently, the solution was filtered and collected residue was washed thrice with absolute alcohol, before being dried at 90 °C and dehydrated in an oven (Deng Yng, Taipei, Taiwan) for 12 h. For preparation of PDA-coated CS cements, the dried powder was mixed and dissolved in distilled water at a liquid/powder ratio of 0.3. The paste was then placed into a Teflon mold of 6 mm diameter and 3 mm height, and further set to shape by placing the specimen in an incubator with 100% relative humidity, at 37 °C for 24 h.

### 2.2. Setting Time and Injectability

The setting times of various PDA-coated CS cements were analyzed according to standards set by the International Standards Organization (ISO) 9917-1. The powder was mixed with distilled water and put into a Teflon cylindrical mold at 37 °C with 100% relative humidity for hydration. Then, the final setting time was considered if the Gilmore needle failed to make a notch with a depth more than 1 mm in six different areas. In addition, the injectability of PDA-coated CS composites were evaluated by the ratio of weighting composites, prior and post injection [19]. Then 4.0 g of pre-mixed paste was loaded into a 6 mL syringe with the 12 G needle by hand press, suggesting that it possessed an even slightly lower bias than an injection of press machine with a preset load. The un-setting PDA-coated CS cement was extruded from the needle, until it was unable to be injected after being incubated at 37 °C in 100% relative humidity at various time-points. The weight of the paste injected through the syringe was measured immediately. The injectability was considered as: I = (m injected/m initial) × 100%, where I is the injectability, and m injected and m initial, are the weights of the materials injected through the syringe and the materials initially contained in the syringe. These results were the average of six specimens performed for each group.

### 2.3. Physicochemical Properties

For this study, the water contact angle for each sample was taken and measured at room temperature. Firstly, the set-materials were placed on top of the platform, and 50 μL Dulbecco’s Modified Eagle Medium (DMEM) was pipetted onto the top of the specimens. After 30 s, close up images of the specimens were taken with a camera and further analyzed using ImageJ from the National Institutes of Health, to measure for the water contact angle of each specimen. For this study, electron spectroscopy (ESCA, PHI 5000 VersaProbe, ULVAC-PHI, Kanagawa, Japan) was used to analyze the chemical components and elemental compositions of the various groups of PDA-coated CS cements, and results of this analysis was presented as atomic percentages. In addition, an X-ray diffractometer (XRD; Bruker D8 SSS, Karlsruhe, Germany) was used to analyze the phase compositions of the various groups of PDA-coated CS cements, and the parameters of this test were set at 30 kV and 30 mA with a scanning speed of 1°/min. Lastly, field emission scanning electron microscopy (FESEM; JEOL JSM-7401F, Tokyo, Japan) was used to observe the morphology and characteristics of the specimens’ surfaces, and the parameters for this test were set in the lower secondary electron image (LEI) mode, at a 3-kV acceleration voltage.

### 2.4. Immersion Behavior

To observe for the various behaviors of specimens after immersion, all specimens were immersed in DMEM solutions at 37 °C, for different time durations. When placed under static immersion, the samples exhibited no observable changes. After 3 and 24 h of immersion in a shaker water bath, the specimens were removed and a scanning electron microscope (SEM; JSM-6700F, JEOL, Tokyo, Japan) was used to observe the microstructures of the specimens, and the parameters for this test were set in the lower secondary electron image (LEI) mode, at a 3-kV acceleration voltage. In addition, an EZ-Test machine (Shimadzu, Kyoto, Japan) was used to test for the diametral tensile strength of the specimens, and this test was done at a loading rate of 1 mm/min. The load deflection curves were used to record the loading weight of the specimens, and the maximal loading weight was obtained from the curves. For this study, each of the eight specimens underwent load bearing tests at various time points.

### 2.5. Cell Adhesion and Proliferation

Prior to the in vitro study, all specimens underwent sterilization by being immersed in 75% EtOH, and subsequently exposed to ultraviolet light for 20 min. The cells used in this study, namely the Wharton’s Jelly mesenchymal stem cells (WJMSC), were from the Bioresource Collection and Research Center (BCRC, Hsin-Chu, Taiwan), and were grown to passage 4 through 7 in mesenchymal stem cell medium (Sciencell). Following which, the cells were removed and cultured directly onto the cement specimen at a cell density of 10^5^ cells per specimen in a 48 cultured-well and incubated with DMEM in an incubator at 37 °C and 5% CO_2_ atmosphere, for various time durations. PrestoBlue^®^ (Invitrogen, Grand Island, NY, USA) was used to evaluate cell viability following the manufacturer’s instructions. Briefly, the specimens were washed with cold phosphate buffered saline (PBS) thrice, prior to removal of DMEM. After which, each well was filled with the PrestoBlue^®^/DMEM mixed reagent (ratio of 1:9), and incubated at 37 °C for 90 min. Subsequently, the incubated reagent from each specimen was transferred to a new 96 well plate, and a Tecan Infinite 200^®^ PRO microplate reader (Tecan, Männedorf, Switzerland) was used to measure the absorbance of the reagent with the wavelength determined at 570 nm, with a reference wavelength of 600 nm. For this study, neat WJMSCs cultured on a plate were used as the control group (Ctl). The results were obtained in triplicate from three separate experiments and averaged.

### 2.6. Cell Morphology

After culturing for 3 h and 24 h, the medium was removed, and the specimens were washed thrice with cold PBS, before fixation with 4% paraformaldehyde for 20 min, and subsequently permeabilized with 1% Triton X-100 for 20 min. After which, the cells were stained with a red F-actin AlexaFluor-594-conjugated phalloidin stain for 60 min, and a green nucleus DAPI stain (4′,6-diamidino-2-phenylindole, dilactate) for 20 min. The above procedures were done at room temperature. Then, the specimens were washed thrice with PBS, and imaged using a Leica TCS SP8 X white light laser confocal microscope (Leica Microsystems GmbH, Wetzlar, Germany).

### 2.7. Ion Released and ECM Secretion

After culturing for 3, 6, and 12 h, the incubated medium were extracted and an inductively coupled plasma-atomic emission spectrometer (ICP-AES; Perkin-Elmer OPT 1MA 3000DV, Shelton, CT, USA) was used to analyze the concentrations of Ca, Si, and P ions released by the various specimens. In addition, collagen I (Col I) and fibronectin (FN) proteins secreted by the WJMSCs cultured on different specimens were also analyzed using a Col I and FN enzyme-linked immunosorbent assay kit (Invitrogen, Carlsbad, CA, USA), and following the manufacturer’s instructions. A standard curve was used to obtain the various concentrations of Col I and FN, and blank cartridges were used as a control for this study. The results were obtained in triplicate from three separate experiments and averaged.

### 2.8. Cell Adhesion-Related Protein

After culturing for 3 h, the cells were lysed using NP40 buffer (ThermoFisher, MA, USA), and a BCA protein assay kit was used to obtain the total protein concentrations from the cell lysates. Next, the cell lysates of 35 μg protein/sample were segregated using sodium dodecyl sulfate-polyacrylamide-polyacrylamide gel electrophoresis (SDS-PAGE), and transferred to a Poly (vinylidene fluoride) membrane (Milipore, Billerica, MA, USA), before further immunoblotting procedures. After which, the transferred proteins were fixated using 2% BSA in TBST for 1 h, before immunoblotting for 2 h with the following antibodies: Primary anti-integrin β1, β-actin (GeneTex, San Antonio, TX, USA), anti-focal adhesion kinase (FAK), and anti-phospho-FAK (p-FAK). Subsequently, the membrane was incubated with horseradish peroxidase (HRP)-conjugated secondary antibodies (ECL detection kit, Invitrogen, Carlsbad, CA, USA) for 1 h, to allow visualization of protein bands using chemiluminescence. The expression levels of the various proteins were normalized to β-actin, for further quantification analysis.

### 2.9. Osteogenesis-Related Genes and Protein Assay

After culturing for 7 and 14 days, the following osteogenesis related genes expressed by WJMSCs were analyzed: Runx2, ALP (alkaline phosphatase), Col I, and osteocalcin (OC). Firstly, TRIzol reagent (Invitrogen, Carlsbad, CA, USA) was used to extract RNA from the various specimens and further analyzed using RT-qPCR. After which, a complementary DNA (cDNA) Synthesis Kit (GeneDireX, Taipei, Taiwan) was used to synthesize cDNA from 500 ng of Total RNA, which were extracted from the above procedure. This was done according to the manufacturer’s instructions, and RT-qPCR primers (Table 1) were selected based on cDNA sequences obtained from the NCBI Sequence Database. For detection, we used SYBR Green qPCR Master Mix (Invitrogen, Carlsbad, CA, USA), and the ABI Step One Plus real-time PCR system (Applied Biosystems, Foster City, CA, USA) was used for target mRNA expressions analysis.

Concurrently, after culturing for 7 and 14 days, the level of alkaline phosphatase (ALP) activity was analyzed using the follow procedures. Firstly, the cells were lysed using 0.2% NP-40, washed thrice with PBS, and then centrifugation was done for 10 min at 2000 rpm. Using p-nitrophenyl phosphate (pNPP, Sigma, St. Louis, MO, USA) as the substrate, ALP levels were determined by first mixing each specimen with pNPP and 1 M diethanolamine buffer for 15 min, followed by terminating of the reaction with 5 N NaOH, and quantification by measuring for absorbances at 405 nm. Lastly, OC protein concentration was obtained using the OC enzyme-linked immunosorbent assay kit (Invitrogen, Carlsbad, CA, USA) by following the manufacturer’s instructions. A standard curve was used to obtain the concentration of OC, and blank cartridges were used as control for this study. All the results from the above studies were obtained in triplicate from three separate experiments and averaged.

### 2.10. Statistical Analysis

The one-way analysis of variance statistical analysis was used to evaluate for significant differences between the data means. For each specimen, Scheffe’s multiple comparison test was used to determine the significance of the deviations, and for this study, all results were considered as significantly different if the data had a *p* value < 0.05.

## 3. Results and Discussion

### 3.1. Characterization of PDA-Coated CS

**Figure 1:**
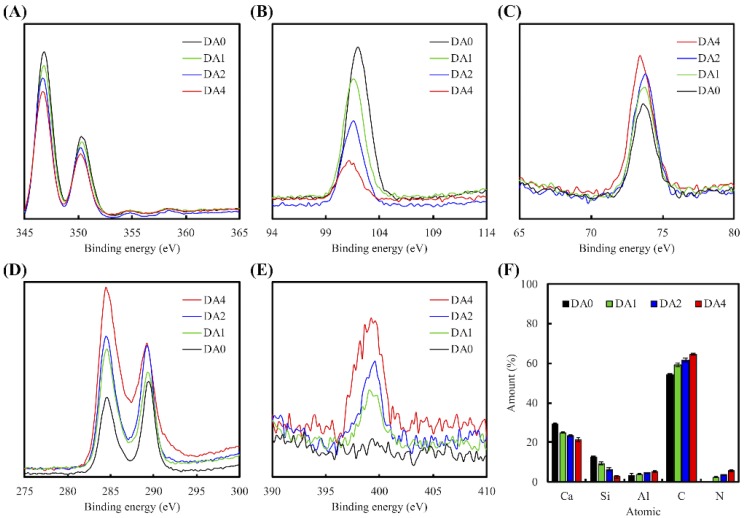
XPS (**A**) Ca2p; (**B**) Si2p; (**C**) Al2p; (**D**) C1s; and (**E**) N2p high-resolution spectra obtained on calcium silicate (CS) cement after coating with dopamine; (**F**) All chemical composition of PDA-coated CS cement.

**Figure 2:**
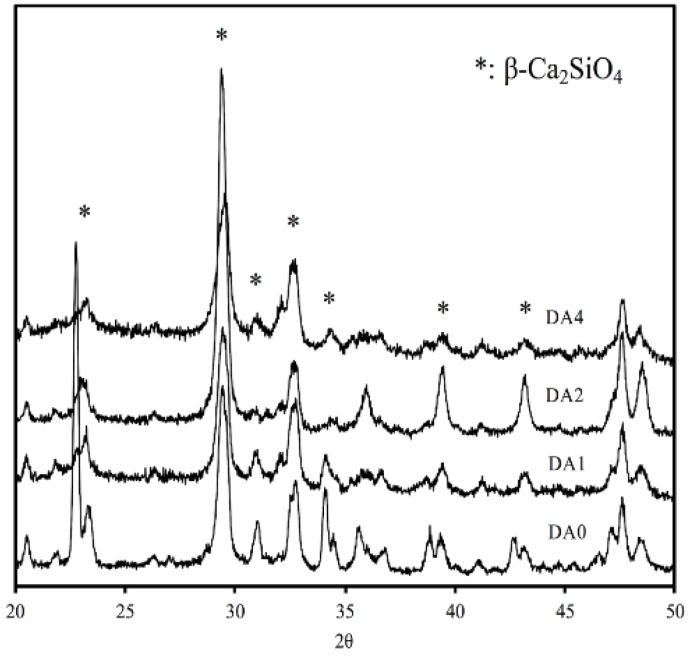
Wide-range XRD patterns of PDA-coated CS cements.

The XPS spectrum of various PDA-coated CS cement is shown in Figure 1. It shows the Ca2p (Figure 1A), Si2p (Figure 1B), Al2p (Figure 1C), C1s (Figure 1D), and N2p (Figure 1E) spectra. There were distinct differences between the composition characteristics of the CS cement, with and without PDA coating (Figure 2F). There was a notable significant increment in the following compounds: Aluminum, carbon, and nitrogen; and notable significant decrement in calcium and silicon. Consistent with reports made by others regarding PDA coated materials, it was observed that the increment of DA coating concentration from 0 mg/mL to 4 mg/mL, led to a decrement of Ca content (29.46 to 21.44%) and increment of C1s (54.37 to 64.66%) and N1s (0 to 5.74%) [18,20]. After the coating process, the C and N contents were much greater than those of pure CS powder, which meant PDA deposition was successful on the powder surface.

As indicated by the XRD results in Figure 2, each specimen shows a primary diffraction peak at 2θ = 29.4°, which correlates to calcium silicate converging with calcite (CaCO_3_). As proven by the secondary diffraction peak 2θ value situated around 32° and 34°, the formation of the first primary diffraction peak was due to the incomplete reaction of the inorganic initiator β-dicalcium silicate (β-Ca_2_SiO_4_) [21]. The ad-mixing of PDA in the calcium silicate bioactive cements, facilitated the newly designed biocomposites. A fast setting time modulation was realized by the amount of PDA accidentally. The setting time of DA0, DA1, DA2, and DA4 was 17.5 min, 17.9 min, 21.0 min, and 24.7 min, respectively.

**Figure 3:**
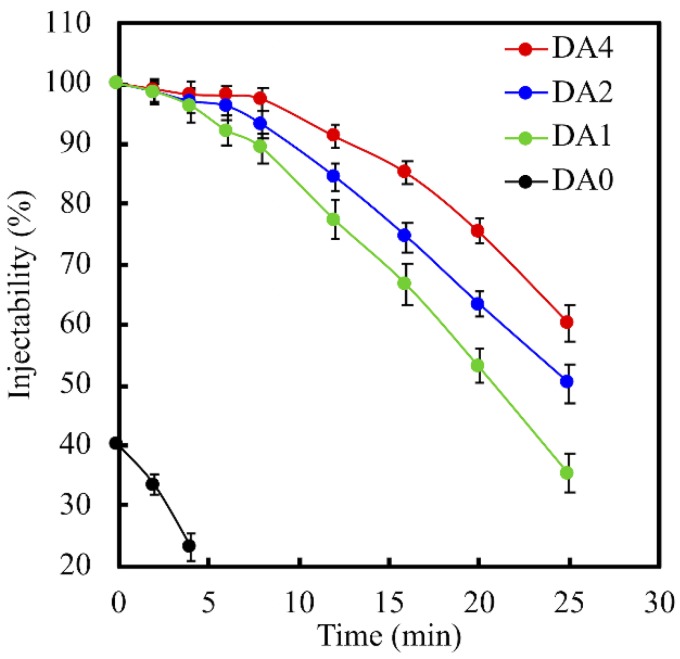
Injectability of various concentration of PDA-coated CS pastes after versus setting time.

The injectability of PDA-coated CS cements is shown in Figure 3. As seen in Figure 3, the injectability is inversely proportionate to the concentration of DA coating. Injectability of biocomposites decreases as DA concentration increases. At 25 min, the injectability of DA0, DA1, DA2, and DA4 was 0%, 35.4%, 50.3%, and 60.4%, respectively. From our study, it was indicated that DA0 had the fastest setting timing, but the lowest injectability. With regards to our previous study, it was recommended that a suitable setting timing for orthopedic applications should be within the range of 10–15 min. We thereby suggest that PDA coating may provide us with more appropriate setting timings, as it had the ability to induce the formation of calcium silicate hydrate.

### 3.2. Morphology and Strength of the Cements after Immersion in DMEM

**Figure 4:**
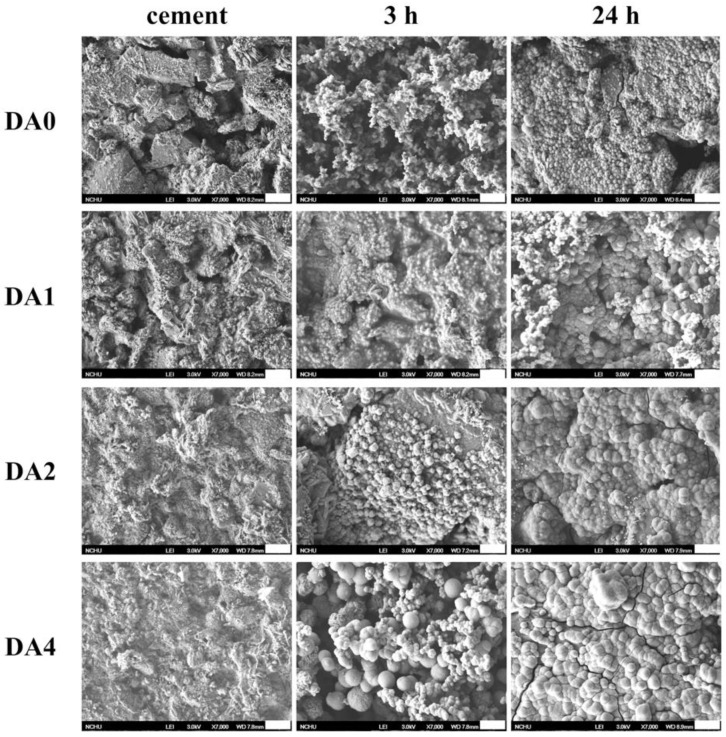
SEM micrographs of different PDA-coated CS cements before and after soaking in DMEM. The scale bar is 2 µm.

Figure 4 showed our pre and post DMEM immersed PDA-coated CS cements SEM micrographs, after 3 and 24 h. The morphological variants before and after the immersion in DMEM for 3 h and 1 day, could be observed. Calcium silicate bioactive cement displayed a looser and rougher surface, in contrast to others having DA addition. DMEM immersion caused precipitation of spherical apatite aggregates in groups with DA coating. This precipitation could be seen from the SEM micrograph images of the specimen’s surfaces after 3 h of immersion. For the morphological changes due to the elevated DA concentration, a more compressed and smoother surface was expected as a result of the adhesion nature of biomimetic mussel molecules. Similarly, Ryu et al. [22] indicated that immersion of PDA coated biocomposites in DMEM allowed induction of apatite formation on surfaces of biocomposites, regardless of their size and structure. The identification of apatite was addressed in the previous section. The size of the spherical granules is proportional to the DA concentration on the surface of the biocomposites. After the biocomposites were immersed in DMEM for 24 h, the bioactive spherical granules were all covered on the biocomposites surfaces, in contrast to the CS cement. These spherical granules apatite crystals were found on the surface of DA2 and DA4 after immersion for 3 h, which was quite different from DA0 and DA2. From our results, there were a higher amount of Ca and P ions chelated on the surfaces of DA4 specimens, as compared to DA0 specimens. From this, we deduced that such chelation increased the quantities of nucleation sites and ionic resources that catered for the higher amount of apatite formation, after immersion in physical solution [17]. In summary, such Ca and P ions chelated PDA coatings were most likely responsible for the increased mineralization on the surfaces of biocomposites. In another study conducted by Wu et al. [23], they showed the mechanism of PDA formation on a β-TCP bioceramic surface, and it was reported that Ca and P ions released from PDA/β-TCP were deposited onto the surface of the material to form a PDA nanolayer/CPC, through the interactions between functional groups of PDA and its ionic products. Therefore, it is reasonable to assume that CS powder was not the only substrate that provided binding surfaces for formation of a PDA nano-layer, and that dissolved Ca, Si ions, and catechol species also played a part in reacting with DA/PDA to form various forms of aggregates, which were dependent on the amount of DA concentration [24]. 

**Figure 5:**
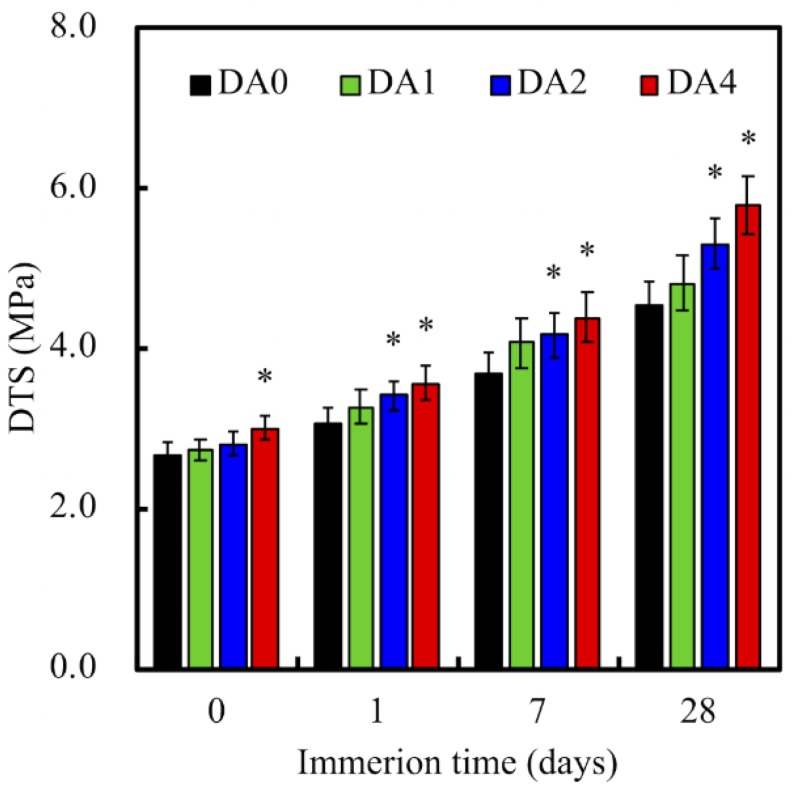
Diametral tensile strength (DTS) of different PDA-coated CS cements, before and after soaking in DMEM. “*” indicates a significant difference (*p* < 0.05) compared to DA0.

Figure 5 indicated the data for our diametral tensile strength tests of our PDA-coated CS cement, after soaking in DMEM. The Diametral tensile strength (DTS) of DA4 showed a significant increase of 13.6% (*p* < 0.05), compared to DA0. As we know, the mechanical strength of the ceramic-based composite strongly depends on the interfacial bonding between powder and powder [25]. As DA4 had a significantly higher ratio of oxygen functional groups present on its surfaces, it was therefore previously suggested that the presence of higher quantities of covalent bonds on its surfaces contributed to the greater tensile strength of the biocomposites [26]. In addition, increased noncovalent bonds, such as hydrogen bonds and Van der Waals interactions etc., further contributed to the tensile strength of the CS cements after hydration [27]. After immersion for 28 days, the DTS of DA2 (5.30 ± 0.31 MPa) and DA4 (5.77 ± 0.36 MPa) was significantly higher than DA0 (4.54 ± 0.29 MPa). Nevertheless, it was further deduced that the concentration of DA coating was directly proportional to the mechanical strength of the biocomposites [27]. It was shown from our study that increasing DA concentration to 4 mg/mL, greatly increased the overall mechanical strength of our final product.

### 3.3. Cell Adhesion

**Figure 6:**
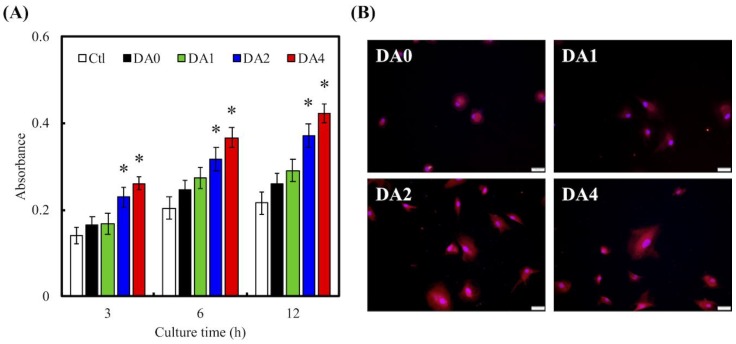
(**A**) PrestoBlue and (**B**) immunofluorescence of Wharton Jelly’s mesenchymal stem cells (WJMSCs) cultured with various specimens for different time points. “*” indicates a significant difference (*p* < 0.05) compared to DA0. The scale bar is 50 µm.

Degree of cellular adhesion was used as a determinant factor in predicting subsequent levels of cellular proliferation and differentiation, with the latter being important factors for determining the amount of tissue growth. Cellular adhesion on the various cements were measured using PrestoBlue assays (Figure 6A). As seen in Figure 6A, throughout the various time culture points, absorbances of both DA2 and DA4 were significantly higher than that of Ctl, DA0, and DA1 (*p* < 0.05). There were no notable differences between DA0 and DA1. The absorbances increased exponentially with the amount of PDA coating, with Ctl having approximately 22%, and DA2 and DA4 having 37% and 42%, respectively, after 12 h of culture. F-actin stains and DAPI immunofluorescence images were used to visualize and observe cell morphology after adhesion and spreading. As seen in Figure 6B, after 12 h of culture, the WJMSCs cultured on DA4 scaffolds exhibited a flattened and elongated morphology, as compared to DA0. In addition, the F-action stains showed cell membrane protrusions and cells were spread over a larger area. WJMSCs in DA0 were in a small spherical shape, which is an indication of a typical non-spreading morphology, indicating that the WJMSCs were not properly adhered to the scaffolds. In addition, there were fewer cells as compared to DA4. These data implied that surface modification with PDA contributed to the fabrication of functional CS substrates, that are non-toxic to cells and able to enhance cellular adhesion, which would influence cellular proliferation and differentiation. This result is consistent with observations made by others, and it was proposed that the quinone group on PDA coating induced a higher amount of protein absorption, such as fibronectin, which promoted cellular adhesion and attachment [21].

### 3.4. Ion Release and ECM Secretion

**Figure 7:**
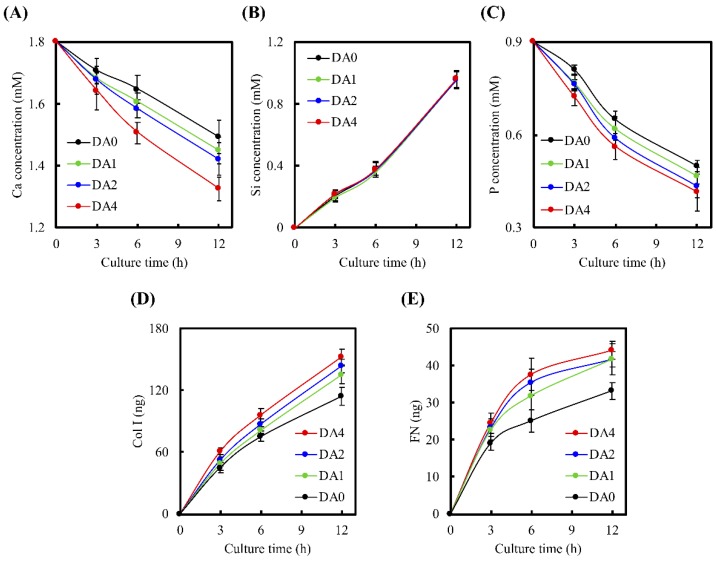
(**A**) Ca; (**B**) Si; and (**C**) P ions concentration released from PDA-coated CS cement after cell seeded for different times; (**D**) Col I and (**E**) FN secreted from the WJMSC after culture for 12 h.

Release of ions from CS scaffolds can also play a role in influencing cell behaviors. Concentrations of Ca, Si, P ions, Col I, and FN secretion from the various specimens after culturing for various duration of time, are depicted in Figure 7. The significant decrement in levels of Ca ions was noted between DA4 and DA0, from approximately 1.50 mM in DA0 to a level of approximately 1.33 mM in DA4, after 12 h of culture (*p* < 0.05). Similarly, P ion concentration decreased for all time points, with DA4 having the lowest concentration of approximately 0.42 mM after 12 h. In the previous study, Ca ions were released from CS-based biomaterials, possibly originating from the less-ordered hydration products, which significantly increased apatite precipitated by increasing local Ca ion concentration. Therefore, the Ca and P ionic product raised the apatite in the surrounding environment and promoted the nucleation behavior of the apatite [28,29,30]. On the other hand, Si ion concentration increased for all groups at all time points. It was further suggested that the secretion of Si from silicate-based materials may play a part in positively influencing cellular activities by means of stimulating production of collagen from cells, which in turn would positively up-regulate growth and differentiation of cells [31]. In addition, appropriate Si ion release of <2 mM may also aid in enhancing proliferation of cells by promoting cellular entry into S and G2 phases [32]. Therefore, our study’s findings of these various ion release profiles may play a role in enhancing cellular behaviors and are of great importance in fabricating functional bone substitutes.

In addition, Col I and FN, which were otherwise known as components of the extracellular matrix produced by cells, are important components for promoting cellular attachment [33]. Col I and FN contain several cell binding substrates, such as RGD motifs, which in turn bind to components of the cell membranes to positively influence cell adhesion [34,35]. These adsorbed proteins on the surface of the scaffold are a provisional matrix for cellular attachment, and different amounts of ECM proteins present, were reported to contribute to various degrees of cellular adhesion [21]. After 12 h of culture, 152.5 ng of Col I and 44.1 ng of FN were measured in the culture medium of DA4, as compared to 114.1 ng of Col I and 33.2 ng of FN in the culture medium of DA0 (Figure 7D,E). WJMSCs planted on DA4 scaffolds exhibited significantly higher Col I and FN secretion, compared to the rest. It is worthy to note that these ECMs are secreted only after initial cellular adhesion and spreading. Our protein secretion profile was consistent, in conjunction with the cellular adhesion and attachment profile shown above. The higher expression of Col I and FN observed on the PDA coated CS scaffolds, indicated that PDA surface modification had the ability to enhance WJMSC attachment and adhesion. In addition, from our previous studies, we proved that hydrolysis was present when CS-based materials were soaked in solutions, and that Si ions further underwent dissolution during incubation [36]. Similarly to the point mentioned above, it was reported by others that the presence of Si ions positively up-regulated cellular behaviors [32]. In addition, Valerio et al. [37] reported that a minimal amount of <2 mM of Si ions from bioglass was able to influence stem cell proliferation and increase ECM production, when compared to the control groups. Following initial cell adhesion on the substrate, WJMSC will stimulate Si ion and secrete ECM components, such as cellular Col I or FN, which adsorbed on PDA-coated CS cement to affect cell behavior [38]. The PDA nanolayer has the ability to be a bridge for covalent immobilization of growth factors and proteins [39]. In summary, the presence of different types and amounts of ECM protein coated on the surfaces of biocomposites, were able to bring about different observed changes in various cellular behaviors [40].

### 3.5. Cell Adhered-Related Protein

**Figure 8:**
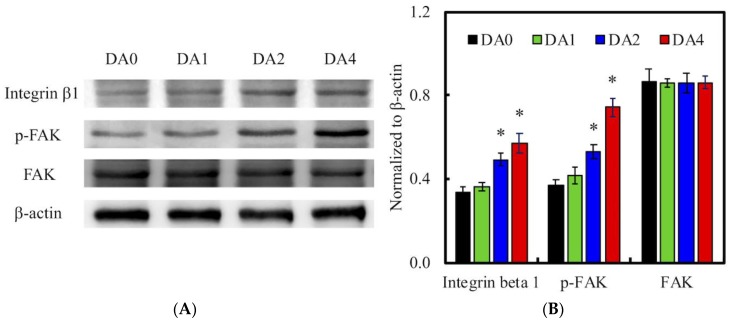
(**A**) Integrin β1 and pFAK expression of WJMSC cultured on various samples after 3 h of incubation; (**B**) The values for the proteins levels were normalized to the actin levels. “*” indicates a significant difference (*p* < 0.05) compared to DA0.

The effect of cell adhesion may be directly related to the improvement of surface hydrophilicity [41] and functional groups, such as OH^−^ and NH_2_^−^ [42]. After seeding for 12 h, the levels of pFAK, FAK, and integrin β1 expression in WJMSCs were examined using western blot (Figure 8). pFAK and integrin β1 expression were significantly higher in DA4 and DA2, than in DA0 (*p* < 0.05). There was an increase of approximately 200% for pFAK expression in cells cultured on DA4, compared to DA0. However, there were no notable significant differences (*p* > 0.05) between DA1 and DA0. This result showed a distinct indication of the determining role of PDA on focal adhesion contacts. Increased cell-ECM interactions trigger cell signaling cascades, which causes up-regulation of growth factor expressions. FAK is activated by binding directly to the cytoplasmic domain of integrin β1 at focal adhesion sites. Studies have identified FAK as an important mediator of integrin signaling, and integrin mediated cell adherence promotes auto-phosphorylation of FAK [43]. After which, pFAK regulates focal-contact formation, cell spreading, and induction of signaling pathways, which are essential for cell motility [34]. pFAK was also reported to further activate extracellular signal-regulated kinase (ERK), which enhances VEGF expression [44]. Therefore, pFAK is often used as a typical biomarker for cell-material interactions, for their important role in cell adhesion complex remodeling. In addition, it was also reported that the blockade of integrin β1 affects FAK and subsequent ERK signaling, and thus decreases cell survivability [45]. Similarly, the increased pFAK and integrin β1 expression in DA2 and DA4, were closely correlated to the increased cellular adhesion and subsequent production of Col I and FN from the results above.

### 3.6. Cell Proliferation

**Figure 9:**
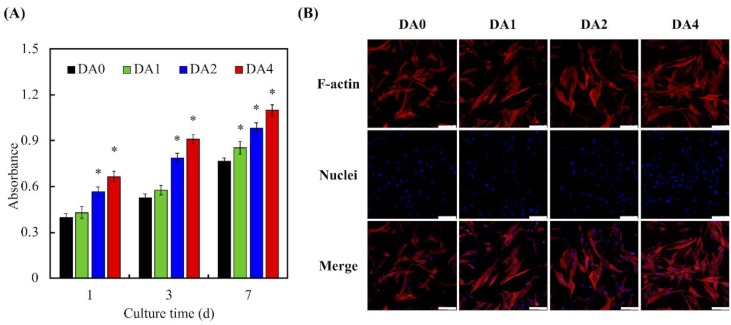
(**A**) PrestoBlue and (**B**) F-actin staining of WJMSC cultured with various specimens for different time points. “*” indicates a significant difference (*p* < 0.05) compared to DA0. The scale bar = 100 µm.

The cell proliferation and fluorescence-stained visual examination of cell F-actin cytoskeleton, which were cultured for a day, are observed in Figure 9. It reveals that cells were spread homogenously on the surface of biocomposites and had the highest degree of spreading, compared to neat calcium silicate bioactive cement. After culture for 1 day, DA4 exhibited the most cell numbers, which was 1.70- and 1.56-times as large as DA0 and DA1, respectively. Interestingly, there were no significant differences (*p* > 0.05) of absorbances ratios (From 3-day to 1-day) between DA0, DA1, DA2, and DA4. We are still unsure of the exact mechanism behind the influences that PDA mediation has on cells. However, it is widely accepted that the surface of hydrophobic materials can be positively modified by depositing a nano-layer of hydrophilic PDA onto the surface. By doing so, we are able to modify the values of critical surface tension to an acceptable range, which can positively enhance biological adhesion or cells through biological methods, such as development of cellular cytoskeleton [46]. From our Prestoblue^®^ assay and cytoskeleton results, it was observed that PDA coating significantly up-regulates WJMSCs proliferation. Even though we are still unsure of the exact mechanism behind this explanation, it can be hypothesized that the ions dissolved and released from our PDA-coated cements were able to positively modulate cell behaviors through the synergistic combinations between the functional groups present in PDA, and the presence of ionic components on the coating surfaces [47,48]. Moreover, we believed that some WJMSC had begun to enter the stage of osteogenesis differentiation, so the growth rate of the cells would gradually slow.

### 3.7. Osteogenic Differentiation

**Figure 10:**
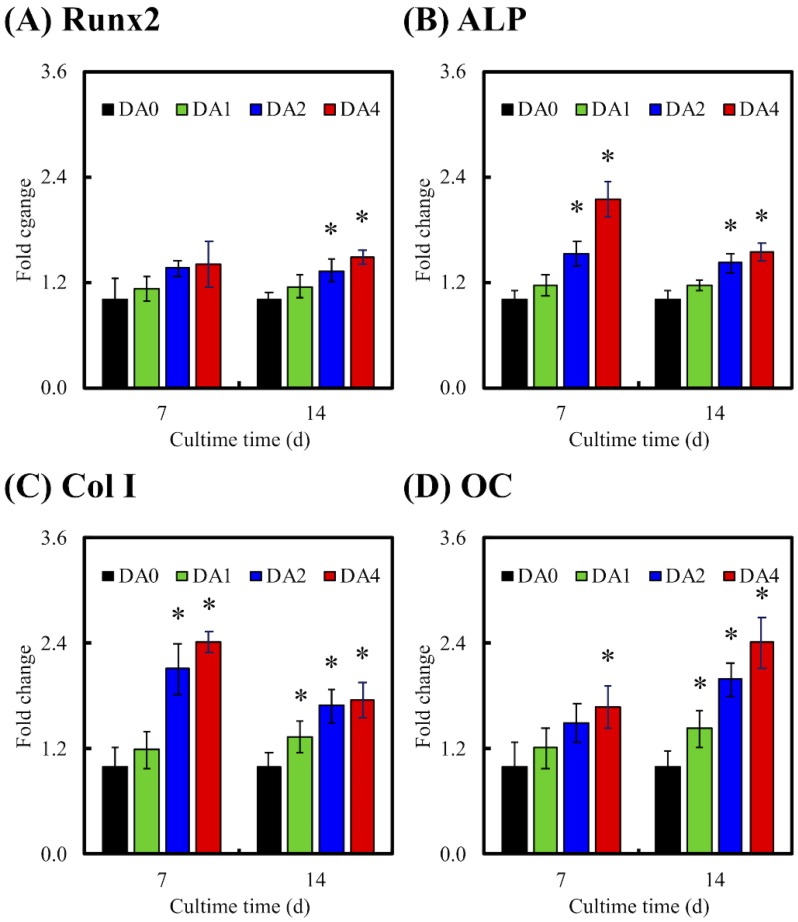
The (**A**) Runx2; (**B**) alkaline phosphatase (ALP); (**C**) Col I; and (**D**) OC gene expression in the WJMSC were cultured on PDA-coated CS cement for 7 and 14 days. “*” indicates a significant difference (*p* < 0.05) compared to DA0.

Osteogenesis related factors were often measured to further determine if there was successful bone formation. In our study, the levels of osteogenesis related genes (Runx2, ALP, Col I, and OC) and osteogenesis related proteins (Col I and OC) were employed as biomarkers, to investigate the levels of cellular differentiation induced by our PDA-coated CS scaffolds (Figure 10). RT-PCR and ELISA were used to investigate gene expression and protein expressions, respectively. Cells were cultured in medium supplemented with osteogenic factors for 7 and 14 days, prior to conduct of tests. It is worthy to note that Runx2, Col I, and ALP were mostly used as an early indicator of osteoblastic lineage, and to study the effects of PDA coating on osteoblast differentiation. Runx2 was reported to be responsible for the induction of mesenchymal stem cells differentiation into osteoblasts, as well as activating several downstream proteins for maintenance of osteoblast differentiation. On the other hand, ALP was well known for its roles in the early mineralization process associated with bone formation, with its level peaking during the early bone formation phases. Col I is mostly expressed in the cell proliferative and ECM secretion stage, whilst ALP is mostly expressed during the post proliferative ECM maturation stage. OC is another common marker for bone formation processes. However, OC is usually found in the last stages of the osteogenesis paradigm, where its role was reported to bind to extracellular Ca for further bone matrix development [14]. Therefore, high levels of OC usually correlate to high bone density.

**Figure 11:**
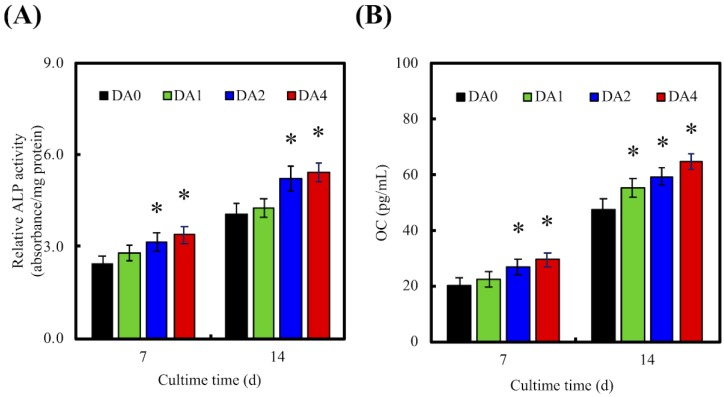
The protein expression of (**A**) ALP and (**B**) OC of WJMSC were cultured on PDA-coated CS cement for different days. “*” indicates a significant difference (*p* < 0.05) compared to DA0.

RT-PCR indicated that cells seeded on DA2 and DA4 scaffolds displayed significant fold changes of Runx2, ALP, Col I, and OC after 14 days of culture. Only DA1 showed significant fold changes of Col I and OC after 14 days of culture. No significant differences between DA1 and DA0 were found for Runx2 and ALP. Respectively, DA2 and DA4 had significant increases in Runx2 expression of 32% and 48%, 41% and 54% significant increases in ALP expression, 69% and 76% significant increases in Col I expression, and 100% and 141% significant increases in OC expression after 14 days of culture. This was further supported by ELISA analysis, demonstrating that cells cultured on DA2 and DA4 had significantly higher protein levels of ALP and OC after 14 days of culture (Figure 11). It is interesting to note that DA4 were able to induce a significantly higher gene expression of ALP, Col I, and OC; and DA2 were able to induce a significantly higher gene expression of ALP and Col I, after 7 days of culture. Similarly, after 7 days of culture, both groups were found to have significantly higher expression of ALP and OC. This data indicated that higher concentration of PDA coating of 2 mg/mL and 4 mg/mL were able to induce higher expression of both gene and protein bone specific biomarkers, allowing the specimens to be more in effective in supporting various cellular functions, such as differentiation and production of extracellular and bone matrixes. In addition, it was reported that higher ALP activity was often correlated to new bone formation [36]. The suitable concentration of PDA-coated on the materials surface was effective in affecting the cell behavior, such as adhesion and differentiation of cells through the production of bone-specific proteins [49]. The OC protein secretion from the WJMSC cultured on DA2 and DA4, was higher than DA0, for 7 and 14 days. Several studies also indicated that PDA-coated substitutes enhanced primary cells proliferation and differentiation [50,51,52]. Lastly, it was found that PDA was often involved in the earliest part of the differentiation process by regulating various growth factors through receptor phosphorylation [53].

Based on our results, we hypothesized two potential mechanisms for the enhancement of WJMSCs behavior when cultured on our PDA nano layer-coated CS specimens. Firstly, PDA coating altered the physical and physiological properties of the surface, such as roughening the surfaces, making it hydrophilic and it provided bioactive functional groups (e.g., −OH, −NH_2_) as promotors, which led to the promotion of cellular adhesion and subsequently increased proliferation and differentiation [23]. Secondly, numerous reports were made regarding the positive effects of mineralization of hydroxyapatite deposits on the surface of biocomposites. Such aggregates were reported to support ECM adsorption, and contributed significantly to the osteogenic and angiogenic differentiation of stem cells. Therefore, with reference to our results, we postulate that the apatite aggregates that had mineralized on the surface may be one of the driving factors behind the enhancement of adhesion, proliferation, differentiation, and bone-related gene expression of WJMSC.

## 4. Conclusions

In summary, we successfully fabricated bio-inspired PDA-coated CS cements via simple immersion methods. This surface modification technique allowed homogenous coating of PDA on the CS surface, as proven by the SEM images and XPS data. Using WJMSC, such modified scaffolds were shown to enhance cell adhesion and promote ECM secretion, such as Col I and FN. In addition, there was a significant increase in the expression of cell adhered-related proteins, such as integrin β1 and pFAK, which in turn led to enhanced proliferation. Furthermore, PDA coatings were also demonstrated to induce osteogenesis and cell differentiation. Therefore, our results supported this simple bio-inspired surface modification of CS cement with PDA, which can be a potential method to regulate and enhance stem cell behavior, serving as an alternative platform for future bone tissue bio-engineering platforms and further research.

## Figures and Tables

**Table 1:** The primer sequences used for the RT-qPCR analysis.

Gene	Forward Primer	Reverse Primer
Runx2	5′-TCAGGCATGTCCCTCGGTAT-3′	5′-TGGCAGGTAGGTATGGTAGTGG-3′
ALP	5′-TCAGAAGCTAACACCAACG-3′	5′-TTGTACGTCTTGGAGAGGGC-3′
Col I	5′-CTGCCCAGAAGAATATGTATCACC-3′	5′-GAAGCAAAGTTTCCTCCAAGACC-3′
OC	5′-GCGCTCTGTCTCTCTCTGACCT-3′	5′-TTTGTAGGCGGTCTTCAAGC-3′
GAPDH	5′-CTCACTCAAGATTGTCAGCA-3′	5′-GTCATCATACTTGGCAGGTT-3′

## References

[1] Qiao H., Tang T. (2018). Engineering 3D approaches to model the dynamic microenvironments of cancer bone metastasis. Bone Res..

[2] Shie M.Y., Chiang W.H., Chen I.W.P., Liu W.Y., Chen Y.W. (2017). Synergistic acceleration in the osteogenic and angiogenic differentiation of human mesenchymal stem cells by calcium silicate-graphene composites. Mater. Sci. Eng. C Mater. Biol. Appl..

[3] Huang S.H., Hsu T.T., Huang T.H., Lin C.Y., Shie M.Y. (2017). Fabrication and characterization of polycaprolactone and tricalcium phosphate composites for tissue engineering applications. J. Dent. Sci..

[4] Schamel M., Bernhardt A., Quade M., Würkner C., Gbureck U., Moseke C., Gelinsky M., Lode A. (2017). Cu^2+^, Co^2+^ and Cr^3+^ doping of a calcium phosphate cement influences materials properties and response of human mesenchymal stromal cells. Mater. Sci. Eng. C Mater. Biol. Appl..

[5] Lin Y.H., Chiu Y.C., Shen Y.F., Wu Y.H., Shie M.Y. (2018). Bioactive calcium silicate/poly-ε-caprolactone composite scaffolds 3D printed under mild conditions for bone tissue engineering. J. Mater. Sci. Mater. Med..

[6] Lai W.Y., Chen Y.W., Kao C.T., Hsu T.T., Huang T.H., Shie M.Y. (2015). Human dental pulp cells responses to apatite precipitation from dicalcium silicates. Materials.

[7] Chen Y.W., Yeh C.H., Shie M.Y. (2015). Stimulatory effects of the fast setting and degradable Ca–Si–Mg cement on both cementogenesis and angiogenesis differentiation of human periodontal ligament cells. J. Mater. Chem. B.

[8] Huang M.H., Shen Y.F., Hsu T.T., Huang T.H., Shie M.Y. (2016). Physical characteristics, antimicrobial and odontogenesis potentials of calcium silicate cement containing hinokitiol. Mater. Sci. Eng. C Mater. Biol. Appl..

[9] Shen Y.F., Ho C.C., Shie M.Y., Wang K., Fang H.Y. (2016). Hinokitiol-loaded mesoporous calcium silicate nanoparticle induce apoptotic cell death through regulation of the function of MDR1 in lung adenocarcinoma cells. Materials.

[10] Lai W.Y., Kao C.T., Hung C.J., Huang T.H., Shie M.Y. (2014). An evaluation of the inflammatory response of lipopolysaccharide-treated primary dental pulp cells with regard to calcium silicate-based cements. Int. J. Oral Sci..

[11] Tu M.G., Chen Y.W., Shie M.Y. (2015). Macrophage-mediated osteogenesis activation in co-culture with osteoblast on calcium silicate cement. J. Mater. Sci. Mater. Med..

[12] Zhong C., Landish B., Zhang C., Cui N., Du J., Lu S., Lin X. (2018). 3D printing hydrogel with graphene oxide is functional in cartilage protection by influencing the signal pathway of Rank/Rankl/OPG. Mater. Sci. Eng. C Mater. Biol. Appl..

[13] Zhai D., Xu M., Liu L., Chang J., Wu C. (2017). Silicate-based bioceramics regulating osteoblast differentiation through a BMP2 signalling pathway. J. Mater. Chem. B.

[14] Huang K.H., Lin Y.H., Shie M.Y., Lin C.P. (2018). Effects of bone morphogenic protein-2 loaded on the 3D-printed MesoCS scaffolds. J. Formosan Med. Assoc..

[15] Liu Y., Ai K., Lu L. (2014). Polydopamine and its derivative materials: Synthesis and promising applications in energy, environmental, and biomedical fields. Chem. Rev..

[16] Cheng Y.L., Chen Y.W., Wang K., Shie M.Y. (2016). Enhanced adhesion and differentiation of human mesenchymal stem cell inside apatite-mineralized/poly(dopamine)-coated poly(ε-caprolactone) scaffolds by stereolithography. J. Mater. Chem. B.

[17] Xu M., Zhai D., Xia L., Li H., Chen S., Fang B., Chang J., Wu C. (2016). Hierarchical bioceramic scaffolds with 3D-plotted macropores and mussel-inspired surface nanolayers for stimulating osteogenesis. Nanoscale.

[18] Kao C.T., Lin C.C., Chen Y.W., Yeh C.H., Fang H.Y., Shie M.Y. (2015). Poly(dopamine) coating of 3D printed poly(lactic acid) scaffolds for bone tissue engineering. Mater. Sci. Eng. C Mater. Biol. Appl..

[19] Chang N.J., Chen Y.W., Shieh D.E., Fang H.Y., Shie M.Y. (2015). The effects of injectable calcium silicate-based composites with the Chinese herb on an osteogenic accelerator in vitro. Biomed. Mater..

[20] Sun X., Cheng L., Zhao J., Jin R., Sun B., Shi Y., Zhang L., Zhang Y., Cui W. (2014). bFGF-grafted electrospun fibrous scaffolds via poly(dopamine) for skin wound healing. J. Mater. Chem. B.

[21] Chen Y.W., Shen Y.F., Ho C.C., Yu J., Wu Y.H., Wang K., Shih C.T., Shie M.Y. (2018). Osteogenic and angiogenic potentials of the cell-laden hydrogel/mussel-inspired calcium silicate complex hierarchical porous scaffold fabricated by 3D bioprinting. Mater. Sci. Eng. C Mater. Biol. Appl..

[22] Ryu J., Ku S.H., Lee H., Park C.B. (2010). Mussel-inspired polydopamine coating as a universal route to hydroxyapatite crystallization. Adv. Funct. Mater..

[23] Wu C., Han P., Liu X., Xu M., Tian T., Chang J., Xiao Y. (2014). Mussel-inspired bioceramics with self-assembled Ca-P/polydopamine composite nanolayer: Preparation, formation mechanism, improved cellular bioactivity and osteogenic differentiation of bone marrow stromal cells. Acta Biomater..

[24] Ball V., Del Frari D., Toniazzo V., Ruch D. (2012). Kinetics of polydopamine film deposition as a function of pH and dopamine concentration: Insights in the polydopamine deposition mechanism. J. Colloid Interface Sci..

[25] Wu Y., Chen M., Chen M., Chen M., Ran Z., Zhu C., Liao H. (2017). The reinforcing effect of polydopamine functionalized graphene nanoplatelets on the mechanical properties of epoxy resins at cryogenic temperature. Polym. Test..

[26] Sharma S., Kothiyal N.C. (2015). Influence of graphene oxide as dispersed phase in cement mortar matrix in defining the crystal patterns of cement hydrates and its effect on mechanical, microstructural and crystallization properties. RSC Adv..

[27] Ma T., Gao H.L., Cong H.P., Yao H.B., Wu L., Yu Z.Y., Chen S.M., Yu S.H. (2018). A bioinspired interface design for improving the strength and electrical conductivity of graphene-based fibers. Adv. Mater..

[28] Mehrali M., Moghaddam E., Shirazi S.F.S., Baradaran S., Mehrali M., Latibari S.T., Metselaar H.S.C., Kadri N.A., Zandi K., Osman N.A.A. (2014). Synthesis, mechanical properties, and in vitro biocompatibility with osteoblasts of calcium silicate-reduced graphene oxide composites. ACS Appl. Mater. Interfaces.

[29] Liu C.H., Huang T.H., Hung C.J., Lai W.Y., Kao C.T., Shie M.Y. (2014). The synergistic effects of fibroblast growth factor-2 and mineral trioxide aggregate on an osteogenic accelerator in vitro. Int. Endod. J..

[30] Zancanela D.C., de Faria A.N., Simão A.M.S., Gonçalves R.R., Ramos A.P., Ciancaglini P. (2016). Multi and single walled carbon nanotubes: Effects on cell responses and biomineralization of osteoblasts cultures. J. Mater. Sci. Mater. Med..

[31] Chen Y.W., Ho C.C., Huang T.H., Hsu T.T., Shie M.Y. (2016). The ionic products from mineral trioxide aggregate–induced odontogenic differentiation of dental pulp cells via activation of the Wnt/β-catenin signaling pathway. J. Endod..

[32] Shie M.Y., Ding S.J., Chang H.C. (2011). The role of silicon in osteoblast-like cell proliferation and apoptosis. Acta Biomater..

[33] Lee J.Y., Chaudhuri O. (2017). Regulation of breast cancer progression by extracellular matrix mechanics: Insights from 3D culture models. ACS Biomater. Sci. Eng..

[34] Shie M.Y., Ding S.J. (2013). Integrin binding and MAPK signal pathways in primary cell responses to surface chemistry of calcium silicate cements. Biomaterials.

[35] Hung C.J., Hsu H.I., Lin C.C., Huang T.H., Wu B.C., Kao C.T., Shie M.Y. (2014). The role of integrin αv in proliferation and differentiation of human dental pulp cell response to calcium silicate cement. J. Endod..

[36] Shie M.Y., Chang H.C., Ding S.J. (2012). Effects of altering the Si/Ca molar ratio of a calcium silicate cement on in vitro cell attachment. Int. Endod. J..

[37] Valerio P., Pereira M.M., Goes A.M., Leite M.F. (2004). The effect of ionic products from bioactive glass dissolution on osteoblast proliferation and collagen production. Biomaterials.

[38] Yeh C.H., Chen Y.W., Shie M.Y., Fang H.Y. (2015). Poly(dopamine)-assisted immobilization of Xu Duan on 3D printed poly(lactic acid) scaffolds to up-regulate osteogenic and angiogenic markers of bone marrow stem cells. Materials.

[39] Lee G.H., Makkar P., Paul K., Lee B. (2017). Development of BMP-2 immobilized polydopamine mediated multichannelled biphasic calcium phosphate granules for improved bone regeneration. Mater. Lett..

[40] Tu M.G., Ho C.C., Hsu T.T., Huang T.H., Lin M.J., Shie M.Y. (2018). Mineral Trioxide Aggregate with mussel-inspired surface nanolayers for stimulating odontogenic differentiation of dental pulp cells. J. Endod..

[41] Wu C., Zhang Y., Zhou Y.Z., Fan W., Xiao Y. (2011). A comparative study of mesoporous glass/silk and non-mesoporous glass/silk scaffolds: Physiochemistry and in vivo osteogenesis. Acta Biomater..

[42] Liu Z., Qu S., Zheng X., Xiong X., Fu R., Tang K., Zhong Z., Weng J. (2014). Effect of polydopamine on the biomimetic mineralization of mussel-inspired calcium phosphate cement in vitro. Mater. Sci. Eng. C Mater. Biol. Appl..

[43] Duan B., Niu H., Zhang W., Ma Y., Yuan Y., Liu C. (2017). Microporous density-mediated response of MSCs on 3D trimodal macro/micro/nano-porous scaffolds via fibronectin/integrin and FAK/MAPK signaling pathways. J. Mater. Chem. B.

[44] Parsons J.T. (2003). Focal adhesion kinase: The first ten years. J. Cell Sci..

[45] Wu B.C., Kao C.T., Huang T.H., Hung C.J., Shie M.Y., Chung H.Y. (2014). Effect of verapamil, a calcium channel blocker, on the odontogenic activity of human dental pulp cells cultured with silicate-based materials. J. Endod..

[46] Ku S.H., Ryu J., Hong S.K., Lee H., Park C.B. (2010). General functionalization route for cell adhesion on non-wetting surfaces. Biomaterials.

[47] Lee D.J., Lee Y.T., Zou R., Daniel R., Ko C.C. (2017). Polydopamine-laced biomimetic material stimulation of bone marrow derived mesenchymal stem cells to promote osteogenic effects. Sci. Rep..

[48] Lin C.C., Fu S.J. (2016). Osteogenesis of human adipose-derived stem cells on poly(dopamine)-coated electrospun poly(lactic acid) fiber mats. Mater. Sci. Eng. C Mater. Biol. Appl..

[49] Chien C.Y., Tsai W.B. (2013). Poly(dopamine)-assisted immobilization of Arg-Gly-Asp peptides, hydroxyapatite, and bone morphogenic protein-2 on titanium to improve the osteogenesis of bone marrow stem cells. ACS Appl. Mater. Interfaces.

[50] Chien C.Y., Liu T.Y., Kuo W.H., Wang M.J., Tsai W.B. (2013). Dopamine-assisted immobilization of hydroxyapatite nanoparticles and RGD peptides to improve the osteoconductivity of titanium. J. Biomed. Mater. Res. Part A.

[51] Rim N.G., Kim S.J., Shin Y.M., Jun I., Lim D.W., Park J.H., Shin H. (2012). Mussel-inspired surface modification of poly(L-lactide) electrospun fibers for modulation of osteogenic differentiation of human mesenchymal stem cells. Colloids Surf. B.

[52] Shi X., Li L., Ostrovidov S., Shu Y., Khademhosseini A., Wu H. (2014). Stretchable and micropatterned membrane for osteogenic differentation of stem cells. ACS Appl. Mater. Interfaces.

[53] Sinha S., Vohra P.K., Bhattacharya R., Dutta S., Sinha S., Mukhopadhyay D. (2009). Dopamine regulates phosphorylation of VEGF receptor 2 by engaging Src-homology-2-domain-containing protein tyrosine phosphatase 2. J. Cell Sci..

